# Perturbation of neuronal cobalamin transport by lysosomal enzyme inhibition

**DOI:** 10.1042/BSR20130130

**Published:** 2014-01-31

**Authors:** Hua Zhao, Kalani Ruberu, Hongyun Li, Brett Garner

**Affiliations:** *Illawarra Health and Medical Research Institute, University of Wollongong, NSW 2522, Australia; †School of Biological Sciences, University of Wollongong, NSW 2522, Australia

**Keywords:** lysosome, mitochondria, neurodegeneration, subcellular-fractionation, vitamin B_12_, AdoCbl, adenosyl cobalamin, Cbl, cobalamin, cpm, counts per minute, DMEM, Dulbecco’s modified Eagle’s medium, Hcy, homocysteine, HS, human serum, LAMP2, lysosomal-associated membrane protein 2, leupeptin, *N*-acetyl-L-leucyl-L-leucyl-L-argininal, MS, methionine synthase, MeCbl, methyl cobalamin, MMCM, mitochondrial methylmalonyl-coenzyme A mutase, TCA, trichloro acetic acid, TC, transcobalamin, TCR, transcobalamin receptor, VDAC1, voltage-dependent anion channel 1

## Abstract

Cbl (cobalamin) utilization as an enzyme cofactor is dependent on its efficient transit through lysosomes to the cytosol and mitochondria. We have previously proposed that pathophysiological perturbations in lysosomal function may inhibit intracellular Cbl transport with consequences for down-stream metabolic pathways. In the current study, we used both HT1080 fibroblasts and SH-SY5Y neurons to assess the impact that protease inhibitors, chloroquine and leupeptin (*N*-acetyl-L-leucyl-L-leucyl-L-argininal), have on the distribution of [^57^Co]Cbl in lysosomes, mitochondria and cytosol. Under standard cell culture conditions the distribution of [^57^Co]Cbl in both neurons and fibroblasts was ~5% in lysosomes, 14% in mitochondria and 81% in cytosol. Treatment of cells with either 25 μM chloroquine or 40 μM leupeptin for 48 h significantly increased the lysosomal [^57^Co]Cbl levels, by 4-fold in fibroblasts and 10-fold in neurons, and this was associated with reduced cytosolic and mitochondrial [^57^Co]Cbl concentrations. Based on Western blotting of LAMP2 in fractions recovered from an OptiPrep density gradient, lysosomal Cbl trapping was associated with an expansion of the lysosomal compartment and an increase in a subpopulation of lysosomes with increased size and density. Moreover, the decreased mitochondrial Cbl that was associated with lysosomal Cbl trapping was correlated with decreased incorporation of [^14^C] propionate into cellular proteins/macromolecules, indicating an inhibition of Cbl-dependent Mm-CoA (methylmalonyl-coenzyme A) mutase activity. These results add support to the idea that lysosomal dysfunction may significantly impact upon Cbl transport and utilization.

## Abstract 2

Vitamin B_12_ (cobalamin) passes through lysosomes to function inside the cell. We show that lysosomal enzyme impairment causes lysosomal vitamin B_12_ trapping. Conditions such as ageing and Alzheimer's disease involve lysosome dysfunction and may similarly suffer from vitamin B_12_ trapping.

## INTRODUCTION

Vitamin B_12_ [Cbl (cobalamin)] is required for erythrocyte formation and DNA synthesis, and plays a critical role in the maintenance of neuron function [1,2]. In plasma and interstitial fluids, Cbl is bound to TC (transcobalamin). The cellular uptake of the TC–Cbl complex is mediated by the TCR (transcobalamin receptor) on the cell surface. The endocytosed complex is delivered to the lysosome where the Cbl is released subsequent to TC proteolysis, whereas the TCR is recycled back to the cell surface [3]. Once Cbl is transported out of the lysosome, it is converted to either MeCbl (methyl cobalamin) in the cytosol or AdoCbl (adenosyl cobalamin) in the mitochondria. MeCbl is used to transform Hcy (homocysteine) to methionine (Met) via cytosolic MS (methionine synthase), whereas AdoCbl is required for the conversion of Mm-CoA (methylmalonyl-coenzyme A) to Succ-CoA (succinyl-coenzyme A) via MMCM (mitochondrial methylmalonyl-coenzyme A mutase). Succ-CoA then enters the Krebs cycle, after which it is utilized in many pathways including conversion to succinate; that may be used in protein synthesis or in the synthesis of porphyrins. Since, extracellular propionic acid is efficiently taken up into cells, converted to propionyl-CoA, then converted to Mm-CoA (via the sequential actions Mm-CoA racemase and MMCM), the incorporation of [^14^C]-propionic acid into cellular macromolecules such as proteins [that are precipitated by TCA (trichloroacetic acid) *in vitro*] is thus an established clinical and basic research tool for evaluating the Cbl-dependent activity of MMCM [4,5].

The importance of the role that the lysosome plays in the delivery of Cbl to MS and MMCM has been highlighted by the discovery of two inborn errors of Cbl metabolism referred to as *cblF* and *cblJ* [5–7]. These life-threatening conditions are caused by a loss of function in either LMBD1 or ABCD4, lysosomal membrane proteins that normally promote Cbl efflux from the lysosome to the cytosol [5,7]. In *cblF* and *cblJ* subjects, Cbl accumulates in lysosomes and the levels of toxic metabolites Hcy and MMA (methylmalonic acid) increase [5,7].

In a seminal paper describing the defective transfer of lysosomal Cbl to the cytosol in fibroblasts derived from a human patient, chloroquine was also used to retard intralysosomal proteolysis in control fibroblasts in order to model lysosomal Cbl trapping [6]. Interestingly, it is known that the acidic pH of the lysosome also influences the conversion of Cbl from the ‘base-on’ to the ‘base-off’ state, which refers to the interaction of the dimethylbenzimidazole moiety of the Cbl molecule with the central Co atom [8]. The Cbl base-off state is thought to be important for subsequent interactions of Cbl with cytosolic cargo proteins. As chloroquine inhibits lysosomal proteases by disrupting the H^+^ gradient across the lysosomal membrane and thereby neutralizing the normally acidic lysosomal pH [9], it is also possible that chloroquine could at least partly inhibit Cbl intracellular transport by blocking its conversion to the base-off state.

It is therefore clear from the *cblF* and *cblJ* inborn errors of Cbl metabolism that transit through the intracellular lysosomal compartment is a strict prerequisite for Cbl utilization by MS and MMCM [3,10–12]. We have also proposed that a more generalized, pathophysiological impairment of lysosomal function that occurs in various disease conditions, such as Alzheimer's disease, lysosomal storage disorders and in age-related neuronal lipofuscinosis, may similarly impede Cbl release from lysosomes as these conditions are all associated with impaired lysosome function that also often includes loss of the proton gradient and lysosomal membrane damage [13–15].

In the present study, we utilize both fibroblast and neuronal cell lines to address fundamental questions related to lysosomal Cbl transport. Firstly, we assess whether a lysosomal proteolysis inhibitor, that does not operate through neutralizing lysosomal pH, could also lead to a trapping of Cbl in the lysosome; and secondly, we investigate whether lysosomal Cbl trapping induced by proteolysis inhibitors could indeed have an effect on MMCM activity as assessed by [^14^C] propionate incorporation into the cellular TCA-precipitated material.

## MATERIALS AND METHODS

### Materials

Chloroquine (Cat no. C6628), leupeptin (*N*-acetyl-L-leucyl-L-leucyl-L-argininal, Cat no. L2884), TCA (Cat no. 9159), penicillin/streptomycin (Cat no. P4333), and HS (human serum, Cat no. H4522) were purchased from Sigma. [^57^Co]cyanoCbl (10 μCi/ml, Cat no. 06B-430002) and [^14^C] propionate (0.1 mCi/ml, Cat no. 11221750), were from, MP Biomedicals. DMEM (Dulbecco's modified Eagle's medium, Cat no. 12800-017) and 200 mM L-glutamine (Cat no. 25030081) were purchased from Life Technologies. FCS (Cat no. SFBS) was from Interpath. OptiPrep (cell separation media as a 60% solution of iodixanol −5,5′− [(2-hydroxy-1,3-propanediyl)-bis(acetylamino)] bis [*N*,*N′*-bis(2,3-dihydroxypropyl-2,4,6-triiodo-1,3-benzenecarboxamide)) was purchased in a lysosome enrichment kit (Cat no. 89839) from Pierce. All other general laboratory reagents were of the highest quality available and obtained through the standard commercial suppliers.

### Cell culture

Experiments were performed using human neuroblastoma cells (SH-SY5Y, ATCC #CRL-2266) and human fibrosarcoma cells (HT1080, ATCC #CCL-121) obtained from the American Type Culture Collection (ATCC). Cells were cultured in DMEM supplemented with 10% (w/v) FCS, 100 μg/ml penicillin/streptomycin, and 2 mM L-glutamine, at 37°C in a humidified atmosphere containing 5% (v/v) CO_2_. Cells were grown in 175 cm^2^ plastic flasks and metabolically labelled in the presence of 10% HS for 48 h with [^57^Co]cyanoCbl (0.025 μCi/ml; Cat. no. 06B-430002, MP Biomedicals) as described previously [16]. Chloroquine (25 μM) or leupeptin (40 μM) were also added during the final 48 h to increase lysosomal pH/inhibit lysosomal proteolysis where indicated.

### Cell homogenization and fractionation

Cell pellets were prepared using a lysosome enrichment kit (Pierce, Cat no. 89839) and transferred to a ball-bearing cell homogenizer (Isobiotec) and homogenized on ice as described in detail [16]. In brief, nuclei and membranous debris were discarded and the organelle suspension (includes lysosomes, mitochondria and cytosol) was collected and layered onto a discontinuous OptiPrep density gradient. The samples were centrifuged at 145000 ***g*** for 4 h at 4°C using a Sorvall MTX 150 ultracentrifuge and a Sorvall S50ST swinging bucket rotor (Thermo Scientific). After centrifugation, ten fractions were carefully withdrawn from the top of the gradients and the organelles separated from the cytosol via a final centrifugation at 20000 ***g*** for 30 min at 4°C as described previously [16]. Both the organelle and cytosolic components of each of the ten fractions were assessed for [^57^Co]Cbl radioactivity, using a Wallace Gamma Counter (PerkinElmer) and for organelle/cytosolic markers as briefly described. For the organelle fractions, radioactivity in LAMP2- and VDAC1 (voltage-dependent anion channel 1)-positive fractions was assigned as lysosomal and mitochondrial, respectively.

In conditions where neuronal proteolysis was inhibited by leupeptin, the density of lysosomes in two of the eight LAMP2-positive fractions (i.e. fractions 7 and 8) became so similar to the mitochondria that it was not possible to completely separate them. In this case, the cpm (counts per minute) values in those two fractions were estimated based on the LAMP2 optical density and comparison with the closest ‘clean’ LAMP2 fractions (i.e. fractions 5 and 6). After subtraction of the estimated lysosomal cpm in fractions 7 and 8, the remaining cpm was assigned as mitochondrial. Using this method both the leupeptin and chloroquine (where the LAMP2/VDAC1 overlap was not pronounced) treatments gave similar results for lysosomal Cbl levels.

### Western blotting

Isolated cellular fractions containing lysosomes, mitochondria and cytosol were probed for appropriate organelle markers by Western blotting; lysosome, LAMP2 (lysosomal-associated membrane protein 2, Southern Biotech); mitochondria, VDAC1(Abcam); cytosol, β-actin (Sigma) and MS (Abnova). Briefly, samples separated on SDS/12% PAGE gels (Mini-Protean II system (Bio-Rad) at 150 V for 70 min followed by transfer at 100 V for 30 min onto 0.45 μm nitrocellulose membranes using a Mini-Trans-Blot Electrophoretic Transfer cell (Bio-Rad). The membranes were blocked in 5% (w/v) non-fat dried skimmed milk powder in PBS) for 1 h at 22°C and then probed with LAMP2 mouse monoclonal antibody (1:4000), VDAC1 rabbit polyclonal antibody (1:4000) or MS goat polyclonal antibody (1:300) at 4°C for 16 h, followed by incubation with the appropriate horseradish-peroxidase-conjugated rabbit anti-mouse (1:4000, Dako), goat anti-rabbit (1:4000, Dako) and rabbit anti-goat (1:4000, Dako) IgG antibodies for 1 h at 22°C. Blots were rinsed in PBS and proteins were detected using enhanced ECL (Amersham Biosciences). The membranes were exposed to ECL Hyperfilm (Amersham Biosciences), developed, scanned and signal intensity was quantified as integrated optical density using NIH Image software.

### Mm-CoA mutase activity [^14^C] propionic acid incorporation

As an indirect measure of MMCM activity, the incorporation of [^14^C] propionic acid into TCA-precipitated macromolecules such as proteins was evaluated [4,5]. Cells were grown to ~80% confluence in 6-well plates under standard culture conditions or in the presence of either chloroquine (25 μm) or leupeptin (40 μm) for 48 h. The cells were then rinsed with PBS and incubated with [^14^C] propionate (1 μCi/ml) in Puck's saline containing 15% (v/v) FCS and 5 mM glucose for 8 h at 37°C. The cells were then rinsed with PBS and incubated with 5% (w/v) TCA for 10 min at 4°C. The cells were collected with a cell scraper and centrifuged at 6000 ***g*** for 5 min at 4°C. Finally, the supernatants were removed and the pellets were dissolved with 1 M NaOH. The amount of [^14^C] propionate in the cell pellets were measured using a Tri-Carb Liquid Scintillation Counter (PerkinElmer). Protein concentrations in the cell pellets were determined using the BCA (bicinchoninic acid) assay.

### Data analysis

Quantitative data are presented as mean±S.E. of three independent experiments unless stated otherwise. Statistical differences were assessed using the *t* test where *P*<0.05 was considered significant. Pearson's correlation analysis was conducted to assess the potential associations between changes in relative mitochondrial [^57^Co]Cbl levels and [^14^C] propionate incorporation into TCA-precipitated cell pellets. Statistical analyses were performed using SPSS Statistics v19.0.0 (IBM).

## RESULTS

### Chloroquine and leupeptin impair subcellular Cbl transport in HT1080 fibroblasts

**Figure 1:**
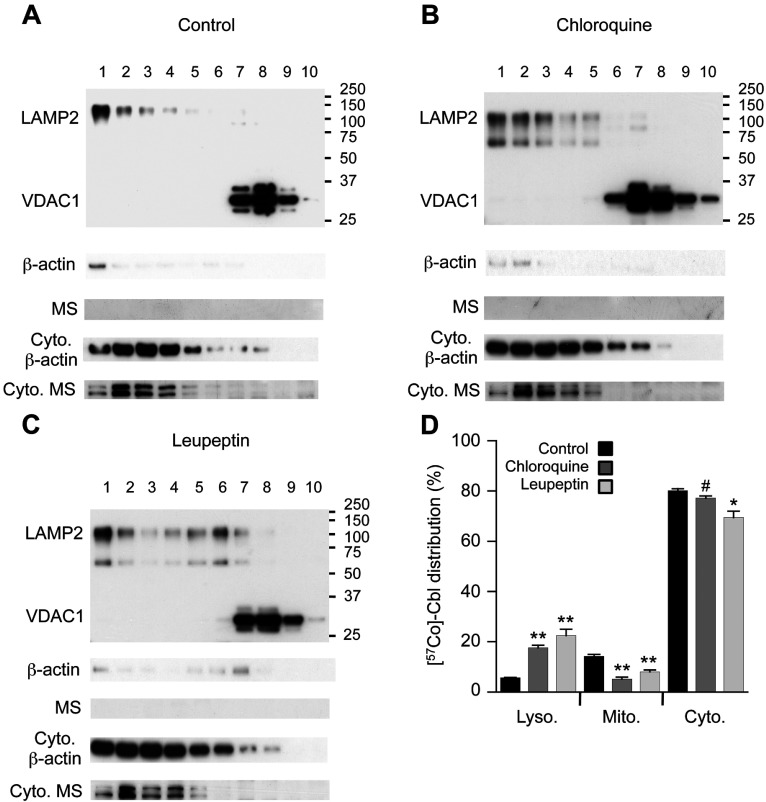
Protease inhibitors increase [^57^Co]Cbl retention in fibroblast lysosomes Approximately 16×10^6^ HT1080 fibroblasts were metabolically labelled with [^57^Co]Cbl for 48 h. The radiolabelled cells were disrupted using a ball-bearing homogenizer and the lysosomal, mitochondrial and cytosolic fractions were separated using an OptiPrep gradient. Intracellular marker proteins LAMP2 (lysosomal), VDAC1 (mitochondrial), β-actin and MS (cytosolic) were then probed by Western blotting in all fractions. Cells were assessed under the standard ‘Control’ culture conditions (**A**) and after the treatment with 25 μM chloroquine for 48 h (**B**), 40 μM leupeptin for 48 h (**C**). The proportional distribution of [^57^Co]Cbl was assessed using a gamma counter and expressed as a percentage of radioactivity in each fraction (**D**). Positions of molecular mass markers (kDa) are shown on the blots. Fractions are labelled 1–10, from lightest (top of gradient) to heaviest (bottom of gradient). Cyto, cytosol. (**A–C**) Data are representatives of three independent experiments. (**D**) Data are means with S.E. represented by the error bars (*n*=3 experiments for each condition).*P<0.05, **P<0.01, #*P*=0.07.

Consistent with previously established methods [16], the separation of fibroblast organelles through an OptiPrep gradient yielded pure lysosomes (LAMP2 positive fractions no. 1–5) and mitochondria (VDAC1 positive fractions no. 7–9) as demonstrated by Western blotting (Figure 1A). The organelle fractions contained only trace amounts of β-actin and were also free of detectable MS, whereas clear signals for both β-actin and MS were detected in the cytosolic fractions.

Previous data indicated that treatment of fibroblasts with chloroquine (25 μM for 21 h) resulted in a dramatic increase in the amount of [^57^Co]Cbl that eluted from a Sephacryl S-200 column in the same position as TC, consistent with a retention of Cbl in lysosomes [6]. In the current study, we directly evaluated the amount of [^57^Co]Cbl in isolated lysosome fractions. Under the standard culture conditions, fibroblast lysosomes contained 5.7% of cellular [^57^Co]Cbl and this was increased 3.1-fold to 17.6% with chloroquine treatment (Figure 1). This retention of [^57^Co]Cbl in the lysosome was associated with a significant 63% drop (from 14.3 to 5.2% of the total cellular levels) in mitochondrial [^57^Co]Cbl and a trend (*P*=0.07) for a 4% drop (from 80.1 to 77.1% of the total cellular levels) in cytosolic [^57^Co]Cbl (Figure 1).

Since chloroquine inhibits lysosomal proteolysis and can theoretically also modulate the transition of lysosomal Cbl to the base-off state (as explained above), we also conducted the similar experiments using another proteolysis inhibitor that does not alter lysosomal pH. We selected the broad specificity competitive transition state inhibitor leupeptin (inhibits cysteine, serine and threonine peptidases) for this purpose. Similar to the results using choroquine, leupeptin treatment also led to a significant 3.9-fold increase in lysosomal [^57^Co]Cbl that was associated with a reduction in both mitochondrial and cytosolic [^57^Co]Cbl levels (reduced by 44 and 13%, respectively; Figure 1).

Interestingly, both chloroquine and leupeptin treatments were associated with the appearance of LAMP2 through a broader range of density fractions isolated from the gradient (compare Figure 1A with Figures 1B and 1C). This may result from both an expansion of the lysosomal compartment and an increase in the size and density of a subpopulation of lysosomes; both of which would be predicted to occur as intralysosomal substrates accumulate [17,18].

### Chloroquine and leupeptin impair subcellular Cbl transport in SH-SY5Y neurons

**Figure 2:**
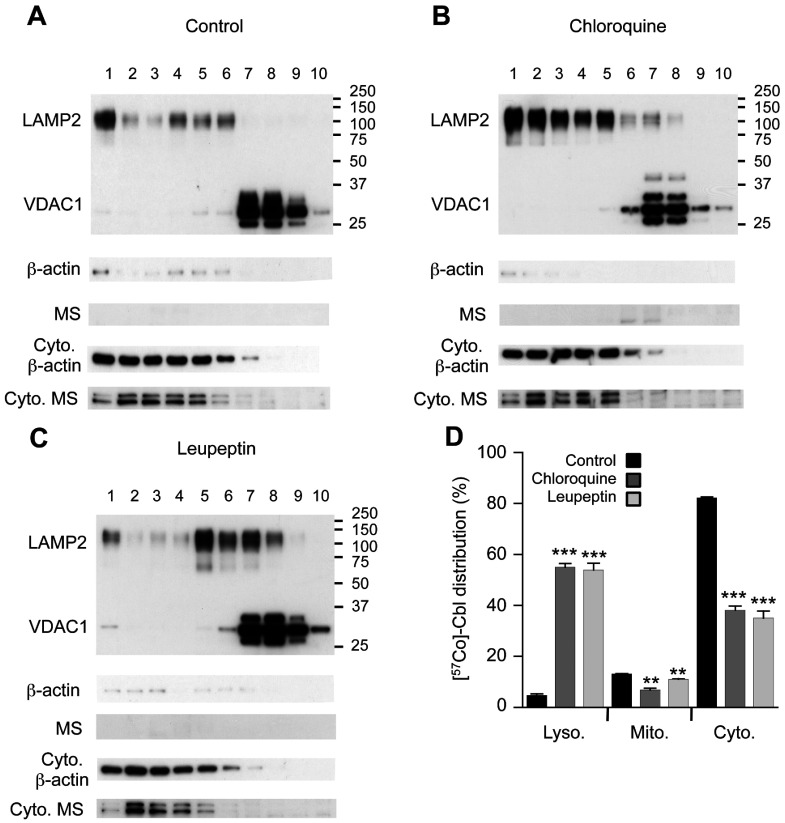
Protease inhibitors increase [^57^Co]Cbl retention in neuronal lysosomes Approximately 16×10^6^ SH-SY5Y neurons were metabolically labelled with [^57^Co]Cbl for 48 h. The radiolabelled cells were disrupted using a ball-bearing homogenizer and the lysosomal, mitochondrial and cytosolic fractions were separated using an OptiPrep gradient. Intracellular marker proteins LAMP2 (lysosomal), VDAC1 (mitochondrial), β-actin and MS (cytosolic) were then probed by Western blotting in all fractions. Cells were assessed under standard ‘Control’ culture conditions (**A**) and after the treatment with 25 μM chloroquine for 48 h (**B**), 40 μM leupeptin for 48 h (**C**). The proportional distribution of [^57^Co]Cbl was assessed using a gamma counter and expressed as a percentage of radioactivity in each fraction (**D**). Positions of molecular mass markers (kDa) are shown on the blots. Fractions are labelled 1–10, from lightest (top of gradient) to heaviest (bottom of gradient). Cyto, cytosol. (**A–C**) Data are representatives of three independent experiments. (**D**) Data are means with S.E. represented by the error bars (*n*=3 experiments for each condition). **P<0.01, ***P<0.0001.

Previous data suggest that age-related lysosomal dysfunction is a particular problem in post-mitotic cells such as neurons [14,19]. We therefore conducted further experiments to assess the impact that lysosomal impairments induced by either chloroquine or leupeptin may have on Cbl distribution in SH-SY5Y neurons. The treatment of neurons with either chloroquine or leupeptin dramatically increased the lysosomal [^57^Co]Cbl levels by ~10-fold in SH-SY5Y neurons and this was associated with a significant ~50% reduction in the cytosolic [^57^Co]Cbl levels (Figure 2). Mitochondrial [^57^Co]Cbl levels were also significantly decreased after either chloroquine or leupeptin treatment (by 48 and 16%, respectively; Figure 2).

In comparing the experiments conducted using fibroblasts and neurons, there were striking differences in the degree of lysosomal accumulation of [^57^Co]Cbl and cytosolic depletion of [^57^Co]Cbl, with both parameters being much more severe in the neuronal cell line. In addition, associated with the more pronounced lysosomal [^57^Co]Cbl accumulation, the change in LAMP2 distribution through the OptiPrep density gradient was also more pronounced in the SH-SY5Yneurons (compare Figures 1B and 1C with 2B and 2C); possibly reflecting a more extensive enlargement of the lysosomal compartment and a greater increase in average lysosome size and density distribution. It was also clear that there were differences in the extent to which mitochondrial [^57^Co]Cbl levels were modulated by chloroquine and leupeptin treatments in the different cell types and this did not necessarily follow the changes in lysosomal [^57^Co]Cbl retention; for example, leupeptin treatment of SH-SY5Y neurons resulted in an approximate 10-fold increase in lysosomal [^57^Co]Cbl and an approximate 50% decrease in cytosolic [^57^Co]Cbl, whereas in the same experimental conditions the mitochondrial [^57^Co]Cbl levels were reduced by only 16% (Figure 2D).

### Lysosomal protease inhibition results in reduced mitochondrial [^57^Co]Cbl that is correlated with impaired MMCM activity

**Figure 3:**
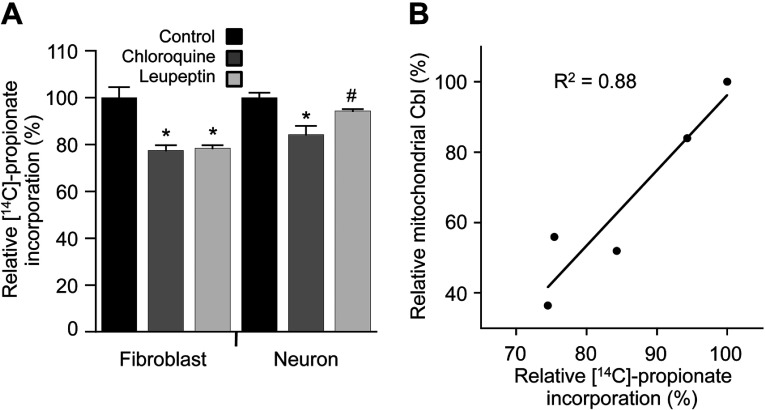
Protease inhibitors reduce cellular [^14^C] propionate incorporation HT1080 fibroblasts and SH-SY5Y neurons were grown to ~80% confluence in 6-well plates and metabolically labelled with [^14^C] propionate under the standard culture conditions or in the presence of either chloroquine (25 μm) or leupeptin (40 μm) for 48 h. The cells were then subjected to 5% TCA and [^14^C] propionate in the cell pellets determined and expressed as a percentage of the control conditions (**A**). Pearson's correlation analysis was conducted to assess potential associations between changes in relative mitochondrial [^57^Co]Cbl levels (derived from the experiments shown in Figures 1 and 2) and [^14^C] propionate incorporation into TCA-precipitated cell pellets (**B**).*P<0.05, #*P*=0.07.

In order to assess whether the variation in mitochondrial [^57^Co]Cbl levels associated with chloroquine or leupeptin treatment was associated with changes in MMCM activity, we used an established method that is based on the incorporation of [^14^C] propionate into TCA-precipitated proteins/macromolecules [4]. Chloroquine treatment resulted in a significant inhibition of [^14^C] propionate incorporation into TCA-precipitated pellets in both fibroblasts and neurons (25.5 and 15.7% reduction, respectively; Figure 3A). Similarly, leupeptin treatment significantly reduced [^14^C] propionate incorporation in fibroblasts (by 24.6%) and there was a trend for reduced [^14^C] propionate incorporation in neurons (5.7%, *P*=0.07; Figure 3A). When the variation in mitochondrial [^57^Co]Cbl levels associated with chloroquine or leupeptin treatment was compared with the relative [^14^C] propionate incorporation values, a significant positive correlation (*R*^2^=0.88, *P*=0.003) was detected (Figure 3B). This suggests that lysosomal entrapment of Cbl may have down-stream consequences on cellular physiology.

## DISCUSSION

In the present work, we have shown that inhibition of lysosomal proteolysis using both pH-dependent and -independent approaches leads to lysosomal Cbl accumulation. Western blotting for LAMP2 in OptiPrep gradient fractions suggested that such lysosomal Cbl ‘trapping’ is also associated with an expansion of the lysosomal compartment and an increase in a subpopulation of lysosomes with increased size and/or density. Moreover, lysosomal Cbl trapping was correlated with decreased incorporation of [^14^C] propionate into cellular proteins/macromolecules; which is a physiological marker of MMCM activity.

Consistent with our previous data [16], only ~6% of cellular [^57^Co]Cbl resides in the lysosome. This was not unexpected as the major sites of Cbl utilization in humans are MS and MMCM, enzymes that are located in the cytosol and mitochondria, respectively. The relatively low level of lysosomal Cbl probably reflects the transient nature of this pool as it is released from the TC–Cbl complex and transported from the lysosome via LMBD1 and ABCD4 [5,7]. Although previous data derived from the cultured human fibroblasts suggest that under control conditions 37.5% of [^57^Co]Cbl resides in lysosomes (fractions from a sucrose gradient of *d* 1.036 to 1.096 g/ml that contained 90.3% of cellular acid phosphatase and 89.6% N-acetly-β-glucosaminidase activity) [6], these earlier studies did not report on possible contamination of lysosomal fractions with cytosol/MS and that may account for the higher values than we report herein. In these earlier studies, patients with a defect in lysosomal release of Cbl were found to accumulate 92.8% of [^57^Co]Cbl in lysosomes, which is a more pronounced lysosomal trapping of Cbl than we detected with the short-term treatment of cells with protease inhibitors. Nevertheless, depending on the cell type examined, we found that increasing the amount of [^57^Co]Cbl in lysosomes from ~6% up to ~20−50% was sufficient to impair Cbl-dependent utilization of [^14^C] propionic acid. Based on these data we speculate that in pathophysiological conditions, that involve lysosomal dysfunction, a similar impairment of Cbl transport may also occur and that could contribute to down-stream changes in Cbl utilization. Examples of conditions that are associated with lysosomal dysfunction (several of which are associated with loss of the proton gradient across the lysosomal membrane) include age-related lipofuscin accumulation [13,19], lysosomal storage diseases [20–22], Alzheimer's disease [23,24] and Parkinson's disease [25–27].

Our results do not allow us to determine whether lysosomal trapping induced by either chloroquine or leupeptin is due to an expansion of the lysosomal compartment or an increase in the amount of Cbl retained in each lysosome. When the [^57^Co]Cbl cpm values were compared with LAMP2 signal in the pure lysosome fractions, we generally observed a close correlation (H. Zhao and B. Garner, unpublished data); however, in conditions where we detected a 10-fold increase in lysosomal [^57^Co]Cbl, we did not observe a change in LAMP2 of the same magnitude. For example, a semi-quantitative comparison of the LAMP2 Western blots (H. Zhao and B. Garner, unpublished data) suggested chloroquine treatment increased LAMP2 levels ~2-fold, whereas the lysosomal [^57^Co]Cbl level was increased ~10-fold (Figure 2). Interestingly, the recent studies suggest that lysosomal protease inhibitors (including chloroquine) have a dual effect on autophagy as they initiate early autophagic processes while suppressing autophagic degradation [28]. This is predicted to result in an expansion of the lysosomal compartment that may be mechanistically related to our current observations. We therefore conclude that Cbl trapping in the lysosome that is induced by protease inhibition is most probably because of a combination of both an expansion of the lysosomal compartment and an increase in lysosomal Cbl concentration.

Another unresolved question that arises from the current study concerns the marked differences in lysosomal [^57^Co]Cbl trapping in fibroblasts as compared with neurons and the associated changes in cytosolic versus mitochondrial [^57^Co]Cbl distribution. In the SH-SY5Y neurons, we observed a very significant drop in cytosolic [^57^Co]Cbl when lysosomal proteolysis inhibitors were present. If the half-life of MS was shorter than the half-life of MMCM, then one might predict a more rapid drop in the [^57^Co]Cbl cytosolic pool; however, the predicted half-lives of these enzymes [29,30] are 30 and 5.5 h, respectively. Therefore the differences in enzyme half-life are unlikely to account for the relative sensitivity of these pools. We are unaware of detailed studies on MS and MMCM turnover in neurons and the impact that Cbl deficiency may have on enzyme half-life but, based on the fact that Cbl deficiency includes a neurological phenotype [31–33], these issues appear to be worthy of future study.

In conclusion, the present study shows that inhibition of lysosomal proteolysis leads to lysosomal Cbl accumulation in fibroblasts and neurons. This lysosomal Cbl trapping appears to be associated with an expansion of the lysosomal compartment and an increase in a subpopulation of lysosomes with increased size and/or density. This is associated with decreased mitochondrial Cbl and an inhibition of MMCM activity as indicated by decreased incorporation of [^14^C] propionate into cellular proteins/macromolecules.
